# Potential Application of SARS-CoV-2 Rapid Antigen Diagnostic Tests for the Detection of Infectious Individuals Attending Mass Gatherings – A Simulation Study

**DOI:** 10.3389/fepid.2022.862826

**Published:** 2022-04-25

**Authors:** Conor G. McAloon, Darren Dahly, Cathal Walsh, Patrick Wall, Breda Smyth, Simon J. More, Conor Teljeur

**Affiliations:** ^1^School of Veterinary Medicine, University College Dublin, Dublin, Ireland; ^2^School of Public Health, University College Cork, Cork, Ireland; ^3^Department of Mathematics and Statistics, University of Limerick, Limerick, Ireland; ^4^School of Public Health, Physiotherapy and Sports Science, University College Dublin, Dublin, Ireland; ^5^Department of Public Health, Health Service Executive West, Galway, Ireland; ^6^Centre for Veterinary Epidemiology and Risk Analysis, School of Veterinary Medicine, University College Dublin, Dublin, Ireland; ^7^Health Information and Quality Authority, George's Court, Dublin, Ireland

**Keywords:** COVID-19, SARS-CoV-2, prevalence, rapid antigen test, predictive value

## Abstract

Rapid Antigen Diagnostic Tests (RADTs) for the detection of SARS-CoV-2 offer advantages in that they are cheaper and faster than currently used PCR tests but have reduced sensitivity and specificity. One potential application of RADTs is to facilitate gatherings of individuals, through testing of attendees at the point of, or immediately prior to entry at a venue. Understanding the baseline risk in the tested population is of particular importance when evaluating the utility of applying diagnostic tests for screening purposes. We used incidence data from January and from July-August 2021, periods of relatively high and low levels of infection, to estimate the prevalence of infectious individuals in the community at particular time points and simulated mass gatherings by sampling from a series of age cohorts. Nine different illustrative scenarios were simulated, small (*n* = 100), medium (*n* = 1,000) and large (*n* = 10,000) gatherings each with 3 possible age constructs: mostly younger, mostly older or a gathering with equal numbers from each age cohort. For each scenario, we estimated the prevalence of infectious attendees, then simulated the likely number of positive and negative test results, the proportion of cases detected and the corresponding positive and negative predictive values, and the cost per case identified. Our findings suggest that for each reported case on a given day, there are likely to be 13.8 additional infectious individuals also present in the community. Prevalence ranged from 0.26% for “mostly older” events in July-August, to 2.6% for “mostly younger” events in January. For small events (100 attendees) the expected number of infectious attendees ranged from <1 across all age constructs of attendees in July-August, to 2.6 for “mostly younger” events in January. For large events (10,000 attendees) the expected number of infectious attendees ranged from 27 (95% confidence intervals 12 to 45) for mostly older events in July-August, to 267 (95% confidence intervals 134 to 436) infectious attendees for mostly younger attendees in January. Given rapid changes in SARS-CoV-2 incidence over time, we developed an RShiny app to allow users to run updated simulations for specific events.

## Introduction

COVID-19 remains a serious threat to public health in Ireland, despite the notably high uptake of vaccination. It thus seems likely that additional nonpharmacological interventions (NPIs) will be necessary to adequately control COVID-19, at least for the near future. However, we would also hope to avoid the more costly NPIs relied on earlier in the pandemic, such as severe limits on movement and gathering, as well as closure of non-essential businesses, schools, and other valuable activities.

In this context, the wider use of rapid antigen diagnostic tests (RADTs) might be useful for helping to control the spread of SARS-CoV-2, and a range of applications have been proposed (1). The potential value of RADTs follows from the fact that they are less expensive, easier to use, and return results much faster than the diagnostic PCR tests underpinning the Irish COVID-19 surveillance and test-and-trace systems (2). There is an important trade off however, as RADTs have both lower specificity (Sp) and sensitivity (Se) to the presence of SARS-CoV-2 than PCR diagnostic tests (3), though there is some evidence to suggest that their ability to detect *infectious* cases could be more favorable (4).

Importantly, the performance of RADTs varies by manufacturer (5) and can be strongly influenced by whether the sample is collected by a trained professional vs. the person undergoing the test (6), and if the latter, whether the sample is collected is with or without supervision (7), which has knock-on effects for costs. Also, as for any diagnostic test, the performance of RADTs *in practice* will be strongly influenced by the prevalence in the tested population. This is because, for a given test sensitivity and specificity, the probability that an individual testing negative is truly uninfected (the negative predictive value, or NPV) will decrease as prevalence increases, while the probability that an individual testing positive is truly infected (the positive predictive value, or PPV) will decrease as prevalence decreases. Given the above, and the fact that the relative costs of false positives vs. false negatives are rarely equal, and can change from context to context, it is important that the cost-effectiveness of RADTs be evaluated for each specific use-case.

One potential application of RADTs is to facilitate gatherings of individuals that might otherwise be prohibited, through testing of attendees at the point of, or immediately prior to entry at a venue, previously described as “testing to enable” (1). Their use in this context has been somewhat evaluated in randomized controlled trials involving RADT based screening of live events [e.g., in Spain (8)]. Such evaluations tended to be conducted when the background prevalence of COVID-19 was relatively low, meaning that the trials were not well-powered to detect impact on SARS-CoV-2 transmission. However, it seems unlikely that similar experiments would be allowed to go forward when the background prevalence is high. Consequently, simulation and/or modeling based approaches might be particularly important for evaluating the use of RADTs in this setting.

Since infection incidence may often differ between age groups at particular points in time, any considerations of the use of RADTs in these contexts need to take into account the age composition of the event. Therefore, the aims of this study were: (1) to estimate the prevalence of infectious individuals within a series of age cohorts by simulating from incidence data and parameters to describe the likely number of infectious days in the population; (2) To simulate mass gatherings with different age-cohort structures to estimate an overall prevalence of infectious individuals; and, (3) to simulate the application of RADTs to these populations to determine the likely utility of these tests at a population level for such events, using case incidence data from two different illustrative time periods.

## Materials and Methods

### Overall Design

With respect to SARS CoV-2, disease occurrence is typically expressed as an incidence reported over a particular time window (e.g., 14-day incidence). However, for the purpose of screening individuals for gatherings, the interest is in the prevalence of infectious individuals at the point in time of the event, rather than the rate at which new infections occur. The prevalence of infectious individuals at any given time point is determined by the incidence of infection as well as the duration of infectiousness for each infected individual. The duration of infectiousness is likely to vary between individuals due to biological variation (9). In addition, given that public health measures are introduced to identify and limit the ability of infected individuals to transmit the virus, the *number of infectious days in the community* for each infected case will vary depending on whether or not they are detected, at what point in the infectious process they are detected and whether or not they heed public health advice to restrict their movements. Furthermore, some individuals may not present for a test (and are therefore not detected) yet may limit their movements based on self-suspicion. Finally, differences in vaccine use and clinical fraction by age (10) mean that many of these factors, and therefore the anticipated number of infectious days in the community for each infected case, will vary by age cohort.

**Figure 1:**
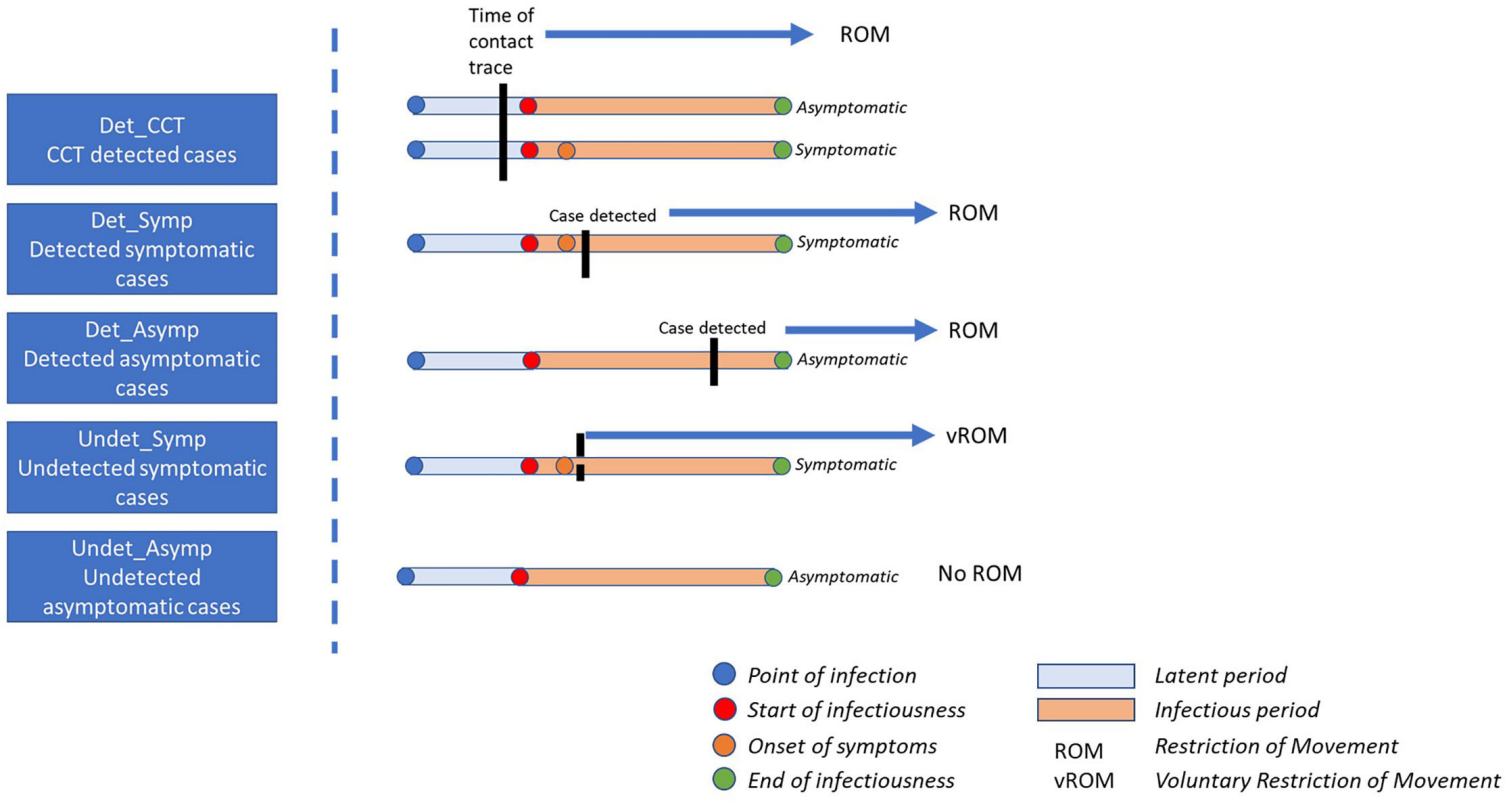
Impact of point of detection for each case category on the number of infectious days in the community. Det_CCT = cases are those that are detected at some point during their infectious window, are documented and reported, and are prospectively identified through contact tracing; Det_Symp, cases that are not identified through contact tracing, are detected, documented and reported, and are symptomatic; Det_Asymp, cases that are not identified through contact tracing, are detected, documented and reported, and are asymptomatic; Undet_Symp, individuals that are never officially detected, documented or reported and are symptomatic, these individuals may, or may not restrict their movement; Undet_Asymp, individuals that are never officially detected, documented or reported and are asymptomatic, these individuals do not restrict their movements.

By modeling disease progression in infected individuals, the timing of testing, and controls associated with contact tracing, we estimated how many undetected infectious individuals there are in the population for each detected (i.e., reported or documented) case on a given day. We then estimated the prevalence of infectious individuals by applying this “multiplier” separately to incidence data from each age cohorts. For each age cohort we assumed that the population consisted of five mutually exclusive infected categories (Figure 1), which differed according to the likely number of infectious days in the community for infected individuals in that category.

First, *detected* cases, that is cases that are reported or documented at any time point in the course of their infectious window, were assumed to consist of three groups: (1) Those that were identified through forward tracing by national contact tracing programmes irrespective of their symptom status (Det_CT); (2) those that *were not* prospectively identified by contact tracing (i.e., were not close contacts of a confirmed case) and were *symptomatic* (Det_Symp), and (3), those that *were not* prospectively identified by contact tracing and were *asymptomatic* (Det_Asymp). Second, it is also recognized that the true number of SARS-CoV-2 infections exceeds those that were detected and documented (11). We assumed that these undetected cases, i.e., those that are never reported or officially documented, could be considered to comprise of two groups: undetected asymptomatic (Undet_Asymp) or undetected symptomatic (Undet_Symp) cases.

We used data from contact tracing to estimate the proportion of detected cases in each cohort. Then using probability distributions to represent uncertainty and variability in key parameters determining the number of infectious days in the community, we simulated the prevalence of infectious individuals for a given 14-day incidence within each age cohort. Gatherings were then simulated with different age cohort structures by drawing from the prevalence distribution of each age cohort to estimate the overall prevalence of infectious individuals at the event. Finally, using probability distributions to model diagnostic test sensitivity (Se) and specificity (Sp) for RADTs, we simulated the potential outcome of test applications to these populations in terms of the number of true positives and negatives, number of false positives and negatives, positive and negative predictive values, each with respect to the target condition “infectious”, and the cost per case identified.

For the purpose of this study, incidence data was taken from Irish case data for the 14-day period ending 6th August 2021 (12). Since case numbers were relatively low in Ireland at that time point (385.5 cases per 100,000 population), a second set of simulations were conducted for the 14-day period ending on the 15th January 2021 (1,533.5 cases per 100,000 population), a period of relatively high levels of infection (12). In addition, to facilitate running simulations for specific purposes, we also developed an R Shiny user interface to allow users to update incidence and proportion of attendees from each age cohort.

### Estimating the Proportion of Individuals in Each “Case Category”

The reported or documented case count of SARS-CoV-2 infections reflects the number of individuals in which the virus has been detected. Individuals may be referred for a test due to contact tracing (i.e., after being identified as a close contact of a confirmed case) or for other reasons (e.g., presentation of symptoms, mandatory testing due to travel). In each case, there was a likelihood that the individual may have been infectious for a number of days prior to being test detected. Depending on the circumstances, the individual may or may not have been observing self-isolation or quarantine.

Contact tracing is used to interrupt chains of transmission. In Ireland, information on the most likely source, date of last contact and the date of the onset of symptoms are recorded for each individual identified as infected (13). Using previous data collected from contact tracing data (14), we calculated the proportion of detected (reported) cases that were previously identified as close contacts of a confirmed case, as Det_CT. For the remaining detected cases, we calculated the proportions that had symptoms, or those that were asymptomatic at the time of contact tracing as Det_Symp and Det_Asymp respectively.

Estimates of the proportion of cases that remain undetected vary across different studies. In reality, changes to the intensity of testing resulting in variation in case ascertainment over time and between regions are likely to impact on these estimates. Mahajan et al. (15) estimated that 35% of infections were likely detected in the US (data until November 2020) (15), whereas other studies from Europe estimated figures of 40% (11) and 52% (16) from Austria and Italy respectively. In Ireland, three serological studies have been conducted to estimate the proportion of undetected cases, with estimates of the fraction of cases detected ranging from 24 to 56% (17–19). Those studies are typically intended to determine the fraction of the population that have developed antibodies indicating exposure to the disease. Frequently those studies also collect information on diagnosed illness of symptoms consistent with the disease. By combining data on sero-prevalence and the diagnostic test accuracy of the antibody tests, the proportion of cases that are undetected can be inferred. For this study, data from the SCOPI study (19) were used in conjunction with a published systematic review of the diagnostic test accuracy of SARS-CoV-2 antibody tests (20) to generate an estimated detected fraction of 50%. We assumed that the majority of these undetected cases were asymptomatic (with no restriction of movement), with the remainder being un-notified/undetected symptomatic cases.

### Scenario Simulation – Estimating the Number of Infectious Individuals Per Event

We simulated a number of different gathering events. We used 3 different event gathering sizes: 100 individuals, 1,000 individuals and 10,000 individuals. For each, we simulated 3 different age cohorts: (1) “Homogenous population” where equal numbers were drawn from all age cohorts; (2) “Mostly younger” events where 50% of attendees were in the 18–24 years age group, 25% were from 0 to 18 age group, 25% were from 25 to 39 age group with no attendees older than 40; “Mostly older” age events where the majority (50%) of attendees were in the 40–65+ age group, 25% were from the 25–39 age group, 25% were older than 65 with no attendees aged 39 or younger.

Using reported incidence, we partitioned cases into each of the categories in Figure 1, and based on the variables in Table 1, created a probability density function for the prevalence of infectious individuals within each age cohort. Next, according to size and age makeup of the event, we drew from each of these distributions to generate an overall number of infectious attendees for each scenario, on each iteration. This process was repeated for 10,000 iterations to capture uncertainty and variability in each of the input parameters.

**Table 1:** Parameters and corresponding distributions used in the simulation model.

**Parameter**	**Mean**	**2.5th – 97.5th percentile**	**Distribution (parameter 1, parameter 2)**	**Source**
Latent period	3.7	1.3–8.3	Lognormal (1.2, 0.464)	(21, 22)
Pre-symptomatic infectious period	2.4	0.4–7.9	Lognormal (0.59, 0.75)	(22, 23)
Post-symptomatic infectious period	7.1	2.8–11.5	Weibull (3.49, 7.90)	(23, 24)
Delay from exposure to contact trace	5.5	1.0–12.0	Sampled from observed distribution	Distribution from Irish contact tracing
RADT Se	0.525^*^	0.437–0.611	Beta (65.31, 59.18)	(25)
RADT Sp	0.999^*^	0.990–1.000	Beta (456.67, 1.46)	(25, 26)

* *Mode*.

### RADT Characteristics

The anticipated number of true positives, false positives, true negatives and false negatives (with target condition “infectious”) was then calculated by simulating from distributions of sensitivity and specificity of the RADTs. Estimates of the sensitivity and specificity of RADTs for the detection of SARS-CoV-2 infection vary considerably (5, 25). Much of this variation may be attributed to differences in the definition of the target condition. For example, in a large screening study in the UK, the reported test sensitivity changed from 0.40 to 0.91 when the target condition was changed from PCR positive, to PCR positive with a cycle threshold <18.3 (27).

For the purpose of this study, we assumed that the majority of cases would be asymptomatic and therefore used the asymptomatic subgroup meta-analysis from Brümmer et al. (25). The sensitivity of the RADT was therefore simulated using a beta distribution with mode of 0.525 and lower 2.5th percentile bound of 0.437 (25). This estimate is similar to a recent report from the use of RADTs in food processing workers in Ireland (26).

Brümmer et al. (25) reported specificity of RADTs >0.990 across different subgroup analyses (25). In contrast, a recent study of supervised sampling of asymptomatic individuals in meat processing plants in Ireland reported only 2 false positive results from 5032 samples (0.9996). For the purpose of this study, specificity was simulated using a Beta distribution with a mode of 0.999 and a lower bound 0.990.

To facilitate modification of the proportions of individuals attending the event, we developed an RShiny application allowing decision makers to specify the number of individuals attending the event, the current or likely incidence per age group, the proportion of attendees from each age cohort and to evaluate the impact on the likely number of infectious attendees, number of true and false, positives and negatives. In addition, the R-code used in this study is included as Supplementary Material.

## Results

Based on the simulation model incorporating typical disease characteristics and contact tracing practice, we estimated that for every reported detected case on a given day, there were 13.8 (95% CI: 5.6–28.7) as yet undetected and potentially infectious cases in the community.

**Figure 2:**
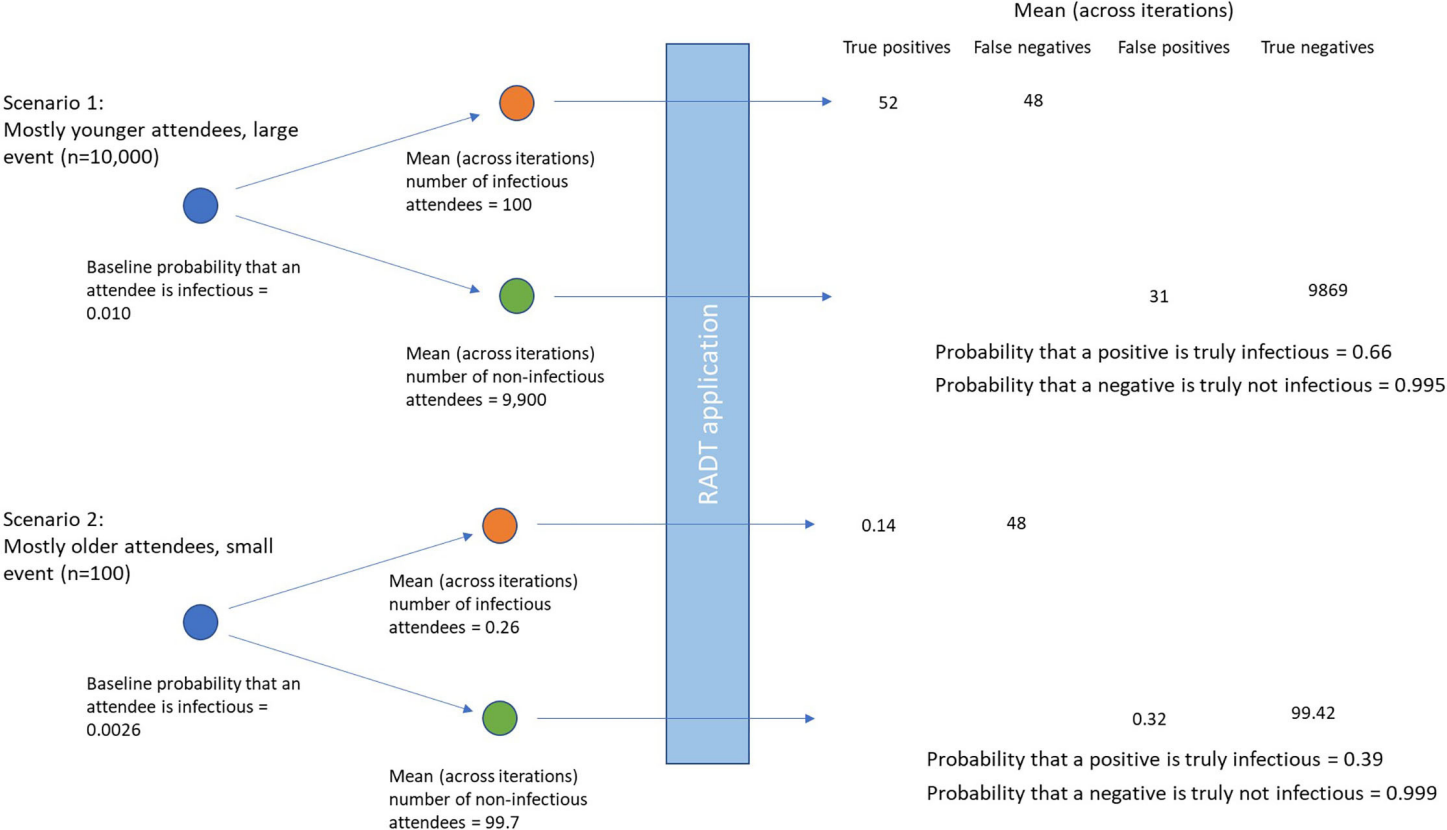
Differences in true and false positives and negatives using two illustrative scenarios of differing prevalences of infectious attendees, and numbers of individuals attending the event.

Results of the simulations of different event types during July-August 2021 are shown in Table 2. Given the differences in incidence by age group, prevalence of infectious individuals at events was highest with “mostly younger” attendees (1.00%), followed by homogenous age gatherings (0.55%) and lowest with “mostly older events” (0.26%). Consequently, the positive predictive value was lowest in the older aged events (0.39, 95% confidence intervals 0.10, 0.86), and was highest in the younger aged events (0.66, 95% confidence intervals 0.29, 0.96). As an example, Figure 2 shows the difference in numbers of true and false positive and negatives across two illustrative scenarios.

**Table 2:** Simulation results from three different congregation scenarios, each under three different event sizes.

**Congregation scenario**	**Prevalence**	**Positive predictive value**	**Event size**	**Mean number of infectious attendees (95% confidence intervals)**	**Mean number of true positives (95% confidence intervals)**	**Mean number of false positives (95% confidence intervals)**	**Mean number of true negatives (95% confidence intervals)**	**Mean number of false negatives (95% confidence intervals)**
Homogenous population	0.0055 (0.0033, 0.0083)	0.5457 (0.2007, 0.9317)	100	0.54 (0, 2)	0.28 (0, 2)	0.32 (0, 2)	99.13 (97, 100)	0.26 (0, 2)
			500	2.77 (0, 7)	1.45 (0, 4)	1.56 (0, 6)	495.66 (490, 500)	1.32 (0, 6)
			10,000	54.95 (30, 88)	28.84 (14, 49)	31.99 (1, 104)	9,913.06 (9,837, 9,958)	26.11 (1, 104)
Mostly younger	0.01 (0.0049, 0.0171)	0.6628 (0.2942, 0.9613)	100	1.01 (0, 4)	0.53 (0, 2)	0.32 (0, 2)	98.67 (96, 100)	0.48 (0, 2)
			500	4.99 (1, 11)	2.61 (0, 7)	1.61 (0, 6)	493.4 (486, 499)	2.38 (0, 6)
			10,000	99.58 (46, 174)	52.23 (22, 95)	31.3 (1, 100)	9,869.12 (9,773, 9,937)	47.35 (20, 86)
Mostly older	0.0026 (0.0015, 0.0041)	0.391 (0.1049, 0.8647)	100	0.26 (0, 2)	0.14 (0, 1)	0.32 (0, 2)	99.42 (97, 100)	0.12 (0, 2)
			500	1.32 (0, 4)	0.69 (0, 3)	1.56 (0, 6)	497.12 (492, 500)	0.64 (0, 6)
			10,000	26.53 (12, 45)	13.92 (5, 26)	31.97 (2, 102.025)	9,941.5 (9,870, 9,978)	12.61 (2, 102.025)

*Prevalence and numbers of true and false positives and negatives relate to infectious attendees. Simulations are based on incidence data from Ireland for the 14-day period ending 6th August 2021*.

For small events (100 attendees) the expected number of infectious attendees was less than or equal to 1 across all age constructs of attendees. For mostly younger events the expected number of infectious attendees was 1.0 and the 95th percentile was 4, whereas for mostly older events the expected number of infectious attendees was 0.26 with a 95th percentile of 2. For large events (10,000 attendees) the expected number of infectious attendees ranged from 26 (95% confidence intervals 12 to 45) for mostly older events, to almost 100 (95% confidence intervals 46–174) infectious attendees for mostly younger attendees.

Given the characteristics of the test, approximately half of these individuals were likely to be detected with the remainder being false negatives. However, there was significant variation with the number of false negatives ranging from 0, 1 for small gatherings of mostly older individuals, to 20–86 false negatives at a large gathering of mostly younger individuals.

For homogenous population events the expected number of false positive attendees was 0.3, 1.6 and 31.3 for small, medium, and large events respectively. However, there was also significant variation with this value, with 95% confidence intervals ranging from 0 to 2 for small gatherings of mostly younger attendees, to between 2 and 101 for large gatherings of mostly older individuals.

The results of applying the model to higher incidence data from 15th January 2021 are shown in Table 3. The prevalence of infectious attendees was higher across all age cohorts, ranging from 1.8% for older events, to 2.6% for younger events. Consequently, the positive predictive value increased to between 0.77 for older, to 0.82 for younger, events.

**Table 3:** Simulation results from three different congregation scenarios, each under three different event sizes.

**Congregation scenario**	**Prevalence**	**Positive predictive value**	**Event size**	**Mean number of infectious attendees (95% confidence intervals)**	**Mean number of true positives (95% confidence intervals)**	**Mean number of false positives (95% confidence intervals)**	**Mean number of true negatives (95% confidence intervals)**	**Mean number of false negatives (95% confidence intervals)**
Homogenous population	0.0185 (0.0122, 0.0263)	0.7775 (0.4782, 0.9805)	100	1.85 (0, 5)	0.96 (0, 3)	0.31 (0, 2)	97.84 (94, 100)	0.89 (0, 3)
			500	9.23 (3, 17)	4.83 (1, 10)	1.54 (0, 6)	489.23 (481, 496)	4.40 (1, 9)
			10,000	185.98 (118, 269)	97.62 (57, 147)	31.11 (1, 100)	9,782.91 (9,681, 9,863)	88.36 (51, 136)
Mostly younger	0.0264 (0.0137, 0.0432)	0.8241 (0.5433, 0.9838)	100	2.63 (0, 7)	1.37 (0, 4)	0.32 (0, 2)	97.05 (93, 100)	1.26 (0, 4)
			500	13.26 (5, 25)	7.00 (2, 15)	1.52 (0, 6)	485.22 (473, 494)	6.26 (1, 13)
			10,000	266.76 (134, 436)	139.84 (68, 236)	30.90 (1, 99)	9,702.34 (9,522, 9,842)	126.92 (61, 218)
Mostly older	0.0181 (0.0105, 0.0279)	0.7699 (0.4554, 0.978)	100	1.80 (0, 5)	0.94 (0, 3)	0.3 (0, 2)	97.9 (94, 100)	0.86 (0, 3)
			500	9.01 (3, 17)	4.71 (1, 10)	1.54 (0, 6)	489.45 (480, 496)	4.3 (1, 9)
			10,000	180.29 (101, 281)	94.64 (50, 153)	31.38 (1, 100)	9,788.33 (9,672, 9,879)	85.65 (45, 140)

*Prevalence and numbers of true and false positives and negatives relate to infectious attendees. Simulations are based on incidence data from Ireland for the 14-day period ending 15th January 2021*.

## Discussion

Understanding the baseline risk in the tested population is of particular importance when evaluating the utility of applying of diagnostic tests for screening purposes. In the context of SARS-CoV-2, case incidence rates are constantly changing. Our study used incidence data to estimate the prevalence of infectious individuals in the community at two particular points in time. However, the methodology can be applied at any time, and therefore has potential as a real-time calculation to support decision making about the control measures required to facilitate mass gatherings while the pandemic is ongoing.

Our findings suggest that for each detected individual on a given day, there are likely to be 13.8 (95% CI: 5.6 to 28.7) additional infectious individuals also present in the community. This “multiplier” includes individuals who will be detected on subsequent days, as well as individuals who will never be detected. Since asymptomatic individuals who will never be detected do not restrict their movement, they contribute a higher number of infectious days in the community than an individual that is detected prospectively through contact tracing for example, thereby contributing more days to this multiplier. However, it is worth noting that these (asymptomatic) individuals are generally considered to have potentially lower infectiousness than symptomatic individuals (28).

To estimate prevalence of infectious individuals from reported case incidence data, we assumed that ~50% of cases were detected. This figure is consistent with the international and national literature (11, 15–19). Undetected cases are a function of numerous factors including testing capacity, disease incidence, practice regarding referral for testing, and asymptomatic disease. In periods of disease surge, testing capacity comes under pressure and tends to be prioritized for symptomatic cases, increasing the proportion undetected. Frontline healthcare workers are generally highly tested because of their increased risk of exposure and to minimize risk too patients, and as such sero-prevalence studies including healthcare workers may under-estimate the undetected fraction. For this analysis, we used an Irish sero-prevalence study that was conducted during June and July 2020 (19). The study may over-estimate the undetected fraction on the grounds that it includes infections in the early stages of the epidemic when testing capacity in Ireland was low. Modeling studies have since shown that there was significant under-ascertainment of cases particularly at the peak of the first wave (beginning March 2020 in Ireland) across many countries (29). Two other Irish sero-prevalence studies have been published: one, conducted in October 2020, found a lower undetected fraction (38%) but was focused on hospital-based healthcare workers (18) and a second, conducted in June to July 2020, that was based in primary care and found a higher undetected fraction (73%) (17). For this analysis, a wide range of uncertainty was adopted for the undetected fraction to reflect the limited data available and the fact that the undetected fraction is likely to vary over time.

We estimated that the expected prevalence of infectious attendees attending simulated events ranged from 0.2 to 1.0% for the simulations based on July-August 2021 case data, and from 1.8 to 2.6% for simulations based on January 2021 case data. For these time periods, this study demonstrates that the prevalence of infectious individuals attending events were higher for events comprised of mostly younger age cohorts. It is worth noting that the impact of infection in these individuals is much lower (30), however, given contact rates within and between different age cohorts (14), higher rates of infection in in younger age groups is likely to lead to increased community incidence and ultimately, transmission to older, higher risk age cohorts.

Interestingly, our study showed that with older events, the positive predictive value of the test was expected to be <40% based on July-August 2021 case data. Estimates of the specificity of RADT tests are generally very high (in excess of 0.99) (25), however even with “higher” prevalence events made of mostly younger individuals, the absolute prevalence of infectious individuals tended to be very low (<1%). Given the low prevalence of infectious individuals, the occurrence of false positives although rare, occur at higher frequencies than true positives leading to the low positive predictive value. However, it is worth noting that for this older age cohort, the impact of infection is much higher than for the other age groups simulated (31), therefore a lower positive predictive value might be tolerated. In contrast, when simulations were applied to time periods with a higher case incidence (January 2021), the resulting positive predictive values were higher, ranging from 0.77 to 0.82.

There are many potential applications of RADT as an aid to the control of SARS-CoV-2 control, Crozier (2021) broadly categorized these as (1) focused asymptomatic testing, (2) focused asymptomatic testing including for example testing for early release from quarantine, and testing to enable otherwise restricted activities, and (3) mass testing. The scenarios modeled in the current study represent only one potential application of the test and should not be used as evidence to support or refute the of RADTs in other contexts (1). Furthermore, a number of limitations are important to note. For the purpose of this study, we used published estimates of the sensitivity and specificity largely informed by a systematic review and meta-analysis (25). As is the case for many test validation studies, these values are based on the use of the test relative to a gold standard. For these studies, PCR is used as a pseudo gold standard. However, PCR itself should not be considered a gold standard test and likely has a test sensitivity which is <100% (32), therefore the true sensitivity of the RADT may be lower than that used for the analysis. It is also recognized, that “reference test” approaches to diagnostic test evaluation will also likely underestimate the specificity of the evaluated test when the reference test is not a true gold standard (33).

On the other hand, a key consideration for such studies is the target condition which the test aims to identify. In our study, we simulated prevalences of *infectious* individuals by considering the duration of the infectious window and likely restriction of movement of infected individuals. However, this target condition does not necessarily align with the test characteristics estimated in conventional test validation studies. A proportion of individuals who have been infected with SARS-CoV-2 remain positive for prolonged periods following initial infection yet are not infectious (34). Therefore, PCR positivity can be considered to have a reduced specificity with regard to a target condition of “infectious,” consequently, RADT test validation studies using PCR as a gold standard will result in downward biased estimates of RADT sensitivity. Whilst it has been shown that transmissibility increases at lower PCR Ct-values (35, 36), and that sensitivity estimates of RADTs substantially increase when the Ct-value threshold of the corresponding PCR test is reduced (25, 26), at present, there does not appear to be consensus on a “safe” Ct-value, above which PCR-detectable cases may be considered non-infectious (35, 36). Estimates of the sensitivity and specificity of any given test will therefore vary according to the distribution of Ct-values within infectious individuals in the sampled population. The distribution used to model RADT test sensitivity in our study can be considered a conservative estimate. Furthermore, for this study, our primary focus was on the prevalence of infectious individuals, however, within this group we did not differentiate between different “degrees of infectiousness” for example between detected and undetected infectious attendees, or between symptomatic and asymptomatic infectious individuals. However, based on literature on this subject to date, it would seem logical to expect that RADT-detected “infectious” individuals would be more infectious, i.e., more likely to result in establishing infection in their close contacts, than RADT-non-detected “infectious” individuals. In some cases, a different test sensitivity may be more appropriate. For example, for an event with relatively young attendees, an increased risk tolerance with respect to infectious attendees may be tolerated. In these cases, it might be reasonable to use a higher sensitivity estimate. Our RShiny application facilitates user inputted test characteristics to run further simulations for specific scenarios, and to assess the impact of uncertainty in test sensitivity and specificity on case detection (https://mcaloon-ucd.shinyapps.io/radts/).

Our analysis also assumed that individuals within age cohorts were independently drawn at random from the overall population. However, it is likely that some degree of clustering is likely to occur with cases, which was not considered in our study. Clustering of cases could result in a disproportionate number of infectious people present at the event, such that realistic range of numbers of infectious individuals potentially attending an event may be greater than those simulated. However, clustering would also imply that those potentially infectious individuals may stay within their grouping and mix less, and therefore it may not lead to markedly increased transmission.

Finally, it is also important to note that the outcomes of the study are conditional on the incidences in a particular region at particular points in time. Simulations based on two illustrative time periods are presented. However, in order to facilitate changing underlying incidence, we developed an RShiny app (https://mcaloon-ucd.shinyapps.io/radts/), to estimate the likely number of infectious event attendees, the estimated number of true and false positives and negatives, as well as the positive predictive value and the cost per case identified.

## Conclusions

Understanding the baseline risk in the tested population is of particular importance when evaluating the utility of applying of diagnostic tests for screening purposes, however incidence data underestimate the risk of infectious individuals at a point in time. This study provides a useful method to estimate the likely number of infectious attendees at a particular event. Whilst the disease characteristics are likely to be similar across countries, contact tracing strategies are likely to change within and between countries over time. However, with some adaptations, this method could be easily applied across countries.

## Data Availability Statement

The original contributions presented in the study are included in the article/Supplementary Material, further inquiries can be directed to the corresponding author.

## Author Contributions

CMA and CT conducted the analyses with advice from DD, CW, BS, and PW. CMA drafted the manuscript. All authors contributed to, reviewed and approved the final manuscript.

## Funding

The contribution of the first author of this study (CMA) was part-funded by the Health Research Board through Evidence Synthesis Ireland [HRB Grant Number CBES-2018-001].

## Conflict of Interest

The authors declare that the research was conducted in the absence of any commercial or financial relationships that could be construed as a potential conflict of interest.

## Publisher's Note

All claims expressed in this article are solely those of the authors and do not necessarily represent those of their affiliated organizations, or those of the publisher, the editors and the reviewers. Any product that may be evaluated in this article, or claim that may be made by its manufacturer, is not guaranteed or endorsed by the publisher.
