# Supplementary material for: Potential Application of SARS-CoV-2 Rapid Antigen Diagnostic Tests for the Detection of Infectious Individuals Attending Mass Gatherings – A Simulation Study
Source: Front Epidemiol. 2022 Apr 25;2:862826. doi: 10.3389/fepid.2022.862826 (PMC10911017; doi:10.3389/fepid.2022.862826)
Supplement: Supplementary file 1 [file Data_Sheet_1.docx]

Supplementary Material

This appendix details the model code used in the study. The code is in two sections. Part 1 describes how the number of infectious people as a multiplier of the incidence was calculated. Part 2 details how gatherings were simulated and tests applied to generate the outcomes detailed in the manuscript. The model was developed in R. The only required packages are ‘*VGAM’* and *‘prevalence’*. In the following appendix text, R commands are in monospace font with grey background. Commands should be run in the sequence given.

# Part 1 - Code used in the calculation of multiplier to determine the number of infectious people in a cohort as a function of the observed incidence

## Model structure

The model simulates cohorts of the population by age group. The user can define the size of the cohort and the number of simulations to run.

# Number of simulations to run

sims=1000

# Population size to simulate

pop=1000

## Definition of model parameters

The model needs a variety of parameters to run, and these are based on published values or observed from Irish epidemiological data. The first set of parameters relate to the timing of an infected individual becoming infectious, and how long they are infectious for.

# Disease progression

# lp = latent period (days) prior to becoming infectious

lp=c(1.2,0.464)

# ip1 = infectious period (days) prior to becoming symptomatic

ip1=c(0.59,.75)

# ip2 = infectious period (days) once symptomatic - assume applies to

# symptomatic and asymptomatic

ip2=c(3.493360,7.903011)

Then we consider the time that typically lapses from someone being a close contact of a confirmed case to when they are alerted to their close contact status and begin to self-isolate. The values of day0.probs relate to the proportion of close contacts that are contacted on day 0, day 1,…, day 12 after exposure. Day zero refers to people who are deemed to be in continuous/ongoing exposure. If those are treated as a day 0, then it implies that they have only just been exposed at the time of contact when in reality they have almost certainly been exposed for several days. For the model we use lag.adjust=TRUE to remove the proportion day zero cases so that they are treated the same as the other close contacts – that is, they were exposed in the days prior to being contacted rather than on the day contacted.

# Lag from exposure to first phone call from contact tracing team (and

# start of ROM)

lag.adjust=TRUE

# Proportion of contacts being contacted on day 0, day 1,…, day 12

day0.probs=c(13.6,9.6,8.5,8.9,10.2,10.4,9.4,7.1,5.8,5.1,4.4,3.9,3.7)

# Ignore those in continuous exposure, assume they have same distribution

# as the rest

if(lag.adjust==TRUE) day0.probs[1]=0

Next we need to define the proportions that are symptomatic test detected, asymptomatic test detected, close contact detected by age group. These have been estimated from the available case detection data in Ireland. These are used to define Dirichlet distributions. The prop.mult variable is used to control uncertainty in these values – we have used a value of 10 which results in uncertainty of approximately ±3%. A value of 1 would result in uncertainty of approximately ±10%

# Proportions of symptomatic test detected, asymptomatic test detected,

# close contact detected by age group

# Age bands used: 00-17, 18-24, 25-39, 40-59, 60+

prop.mult=10

age.data=list(c(20,13,67),c(30.5,12.5,57),c(37.5,17.5,45),c(37.5,13.5,50),c(36.5,15.5,49))

# number of age bands used

n.ages=length(age.data)

We then estimate the proportions of symptomatic undetected and asymptomatic undetected cases.

# Proportions of symptomatic undetected and asymptomatic undetected

unknown.prop.symptomatic=rbeta(sims,2,6)

known.prop=rbeta(sims,6.058,5.991)

unknown.prop=(1/known.prop)-1

unknown.symptomatic=unknown.prop*unknown.prop.symptomatic

unknown.asymptomatic=unknown.prop*(1-unknown.prop.symptomatic)

We also need to take into account the probability of adherence to restriction of movement (ROM) and or self-isolation in detected cases. Here we distinguish between test-detected symptomatic, test-detected asymptomatic and contact tracing-detected cases.

# Adherence to ROM/self-isolation

# test=detected symptomatic

tds.adhere=rbeta(sims,95,5)

# test-detected asymptomatic

tdas.adhere=rbeta(sims,90,10)

# close contact detected

cc.adhere=rbeta(sims,90,10)

Finally we define a matrix to store the simulation outputs – that is, the estimated multiplier for each simulation for each age group.

age.res=matrix(0,nrow=sims,ncol=n.ages)

## Main simulation code

We then run the simulation model, which does the stated number of simulations for each of the defined age bands.

curi=0

simstot=sims*n.ages

pb=txtProgressBar(min=0,max=1,style=3)

for(i.age in 1:n.ages){

known.props=age.data[[i.age]]*prop.mult

sim.props=VGAM::rdiric(sims,known.props)

res=as.data.frame(matrix(0,nrow=sims,ncol=2))

names(res)=c("days","pop")

for(i in 1:sims){

# test detected symptomatic

# assume that they go into ROM on average 1 day after becoming

# symptomatic

tds.pop=round(pop*sim.props[i,1])

infectious.period1=rlnorm(tds.pop,ip1[[1]],ip1[[2]])

infectious.period2a=rlnorm(tds.pop,-0.2160383,0.6573253) #

# mean of one day, upper bound of 3

infectious.period2b=rweibull(tds.pop,ip2[[1]],ip2[[2]])

# total = pre-sympromatic infectious period plus days prior to

# detection (or all days for proportion non-adherent)

test.detected.symptoa=infectious.period1+infectious.period2a

test.detected.symptob=infectious.period1+infectious.period2b

# test detected asymptomatic

# assume that they are detected at a random point post

# becoming infectious [ie they are not detected prior to being

# infectious]

tdas.pop=round(pop*sim.props[i,2])

infectious.period1=rlnorm(tdas.pop,ip1[[1]],ip1[[2]])

infectious.period2=rweibull(tdas.pop,ip2[[1]],ip2[[2]])

# random uniform portion of total unless non adherent

test.detected.asymptoa=infectious.period1+infectious.period2*

runif(tdas.pop)

test.detected.asymptob=infectious.period1+infectious.period2

# close contact detected

# assume they go into ROM based on the defined distribution

cc.pop=round(pop*sim.props[i,3])

latent.period=rlnorm(cc.pop,lp[[1]],lp[[2]])

infectious.period1=rlnorm(cc.pop,ip1[[1]],ip1[[2]])

infectious.period2=rweibull(cc.pop,ip2[[1]],ip2[[2]])

rom.start=sample(0:12,cc.pop,replace=T,prob=day0.probs)

rom.infectious=rom.start-latent.period

rom.infectious[which(rom.infectious<0)]=0

max.infectious=infectious.period1+infectious.period2

cc.detecteda=apply(cbind.data.frame(rom.infectious,

max.infectious),1,min)

cc.detectedb=infectious.period1+infectious.period2

# not detected, symptomatic

# assume they voluntarily go into ROM at some random point

# after becoming symptomatic

nds.pop=round(pop*unknown.symptomatic[i])

infectious.period1=rlnorm(nds.pop,ip1[[1]],ip1[[2]])

infectious.period2=rweibull(nds.pop,ip2[[1]],ip2[[2]])*

runif(nds.pop)

not.detected.sympto=(infectious.period1+infectious.period2)

# not detected, asymptomatic

# assume they do not go into ROM at all

ndas.pop=round(pop*unknown.asymptomatic[i])

infectious.period1=rlnorm(ndas.pop,ip1[[1]],ip1[[2]])

infectious.period2=rweibull(ndas.pop,ip2[[1]],ip2[[2]])

not.detected.asympto=(infectious.period1+infectious.period2)

# total infectious days per cohort

tds.fig=sum(test.detected.symptoa)*tds.adhere[i]+

sum(test.detected.symptob)*(1-tds.adhere[i])

tdas.fig=sum(test.detected.asymptoa)*tdas.adhere[i]+

sum(test.detected.asymptob)*(1-tdas.adhere[i])

cc.fig=sum(cc.detecteda)*cc.adhere[i]+

sum(cc.detectedb)*(1-cc.adhere[i])

undetected.fig=sum(not.detected.sympto)+

sum(not.detected.asympto)

res[i,1]=tds.fig+tdas.fig+cc.fig+undetected.fig

res[i,2]=tds.pop+tdas.pop+cc.pop+nds.pop+ndas.pop

curi=curi+1

setTxtProgressBar(pb, value=curi/simstot)

}

# Multiplier - number of infectious person days per known case

mult=res$days/pop

age.res[,i.age]=mult

}

The age.res matrix contains the estimated multipliers by age group for each simulation. The *fitdistrplus* package can be used to determine a distribution of best fit for the multiplier, or else the simulated values can be used directly as part of a model that simulates infectious people in a cohort as a function of the reported incidence.

# Part 2 - Code used to simulate gatherings of different sizes and age structures and application of diagnostic tests.

## Set input data for the simulations

###multipliers from Part 1

m1 = c(2.5402, 0.4160) ## multiplier for the first age cohort

m2 = c(2.5402, 0.4160)

m3 = c(2.5402, 0.4160)

m4 = c(2.5402, 0.4160)

m5 = c(2.5402, 0.4160)

###Input table for reported 14-day incidence rates per 100000 (based on January 2021 case numbers)

i1 = 674.71

i2 = 3015.33

i3 = 1920.46

i4 = 1752.30

i5 = 1304.18

###Set the proportions of attendees from each age cohort

##even – same number of attendees from each age cohort

p1=0.2

p2=0.2

p3=0.2

p4=0.2

p5=0.2

###mostly younger – all attendees from cohort 2 and 3

#p1=0

#p2=0.5

#p3=0.5

#p4=0

#p5=0

##mostly older all attendees from cohort 3, 4 and 5

#p1=0

#p2=0

#p3=0.25

#p4=0.5

#p5=0.25

###Set test characteristics using mode and 2.5th percentiles

se1=0.525

se2=0.437

sp1=0.999

sp2=0.990

## Create a dataframe with all of the inputs included

input <- data.frame(i1=i1, i2=i2, i3=i3, i4=i4, i5=i5,

p1=p1, p2=p2, p3=p3, p4=p4, p5=p5,

se1, se2, sp1, sp2)

## Main simulation part

This section simulates each of the scenarios for a user-defined number of iterations, stores then summarises those simulations, and creates a final table of results.

##library("prevalence")

## Set number of simulations to run

sims=10000

##current incidence per cohort (per 100k) – convert 14-day to daily

i1 = input$i1/14

i2 = input$i2/14

i3 = input$i3/14

i4 = input$i4/14

i5 = input$i5/14

#convert to prevalence using multipliers from part 1

#need to convert incidence from per 100,000 to per individual

pv1 = c((i1/100000) * qlnorm(p = 0.5, m1[1],m1[2]),

(i1/100000) * qlnorm(p = 0.975, m1[1],m1[2]))

pv2 = c((i2/100000) * qlnorm(p = 0.5, m2[1],m2[2]),

(i2/100000) * qlnorm(p = 0.975, m2[1],m2[2]))

pv3 = c((i3/100000) * qlnorm(p = 0.5, m3[1],m3[2]),

(i3/100000) * qlnorm(p = 0.975, m3[1],m3[2]))

pv4 = c((i4/100000) * qlnorm(p = 0.5, m4[1],m4[2]),

(i4/100000) * qlnorm(p = 0.975, m4[1],m4[2]))

pv5 = c((i5/100000) * qlnorm(p = 0.5, m5[1],m5[2]),

(i5/100000) * qlnorm(p = 0.975, m5[1],m5[2]))

##create prevalence distributions per age cohort

a1 <- as.numeric(betaExpert(pv1[1], upper=pv1[2], p=0.975)[1])

b1 <- as.numeric(betaExpert(pv1[1], upper=pv1[2], p=0.975)[2])

a2 <- as.numeric(betaExpert(pv2[1], upper=pv2[2], p=0.975)[1])

b2 <- as.numeric(betaExpert(pv2[1], upper=pv2[2], p=0.975)[2])

a3 <- as.numeric(betaExpert(pv3[1], upper=pv3[2], p=0.975)[1])

b3 <- as.numeric(betaExpert(pv3[1], upper=pv3[2], p=0.975)[2])

a4 <- as.numeric(betaExpert(pv4[1], upper=pv4[2], p=0.975)[1])

b4 <- as.numeric(betaExpert(pv4[1], upper=pv4[2], p=0.975)[2])

a5 <- as.numeric(betaExpert(pv5[1], upper=pv5[2], p=0.975)[1])

b5 <- as.numeric(betaExpert(pv5[1], upper=pv5[2], p=0.975)[2])

#Create sensitivity and specificity distributions

Se.a = as.numeric(betaExpert(input$se1, lower=input$se2, p=0.975)[1])

Se.b = as.numeric(betaExpert(input$se1, lower=input$se2, p=0.975)[2])

Sp.a = as.numeric(betaExpert(best = input$sp1, lower=input$sp2, p=0.975)[1])

Sp.b = as.numeric(betaExpert(input$sp1, lower=input$sp2, p=0.975)[2])

###Creat list of overall attendance numbers to simulate

nlist = c(100, 500, 10000)

##Create empty data frame to store data from simulations

ov_dat <- data.frame(prev=numeric(), prev_025=numeric(), prev_975=numeric(),

PPV=numeric(), PPV_025=numeric(), PPV_975=numeric(),

nInf=numeric(), nInf_025=numeric(), nInf_975=numeric(),

nTP=numeric(), nTP_025=numeric(), nTP_975=numeric(),

nFP=numeric(), nFP_025=numeric(), nFP_975=numeric(),

nTN=numeric(), nTN_025=numeric(), nTN_975=numeric(),

nFN=numeric(), nFN_025=numeric(), nFN_975=numeric())

for (i in 1:3){

###create data frame in inner loop to store simulations

input$n = nlist[i]

df = data.frame(pop_size=numeric(), nInf = numeric(), nTP=numeric(), nFP=numeric(),

nTN=numeric(), nFN=numeric(), PPV=numeric(),

PV_prev = numeric(), Se=numeric(), Sp=numeric())

for (j in 1:sims) {

n1 = round(input$n*p1)

n2 = round(input$n*p2)

n3 = round(input$n*p3)

n4 = round(input$n*p4)

n5 = round(input$n*p5)

c1_inf <- rbinom(n=1, size=n1, prob=rbeta(1, a1, b1))

c2_inf <- rbinom(n=1, size=n2, prob=rbeta(1, a2, b2))

c3_inf <- rbinom(n=1, size=n3, prob=rbeta(1, a3, b3))

c4_inf <- rbinom(n=1, size=n4, prob=rbeta(1, a4, b4))

c5_inf <- rbinom(n=1, size=n5, prob=rbeta(1, a5, b5))

PV_prev = p1*rbeta(1, a1, b1) +

p2*rbeta(1, a2, b2) +

p3*rbeta(1, a3, b3) +

p4*rbeta(1, a4, b4) +

p5*rbeta(1, a5, b5)

Se = rbeta(1, Se.a, Se.b)

Sp = rbeta(1, Sp.a, Sp.b)

PPV=(PV_prev*Se)/((PV_prev*Se) + (1-PV_prev)*(1-Sp))

nInf = sum(c1_inf, c2_inf, c3_inf, c4_inf, c5_inf)

nTP = rbinom(size=nInf, n=1, prob=Se)

nFP = rbinom(size=(input$n-nInf), n=1, prob=1-Sp)

nTN = (input$n-nInf) - nFP

nFN = nInf - nTP

A=data.frame(nInf, nTP, nFP, nTN, nFN, PPV, PV_prev, Se, Sp)

df <- bind_rows(df, A)

}

dat = data.frame(pop_size=nlist[i],

prev=mean(df$PV_prev), prev_025=quantile(df$PV_prev, 0.025),

prev_975=quantile(df$PV_prev, 0.975),

PPV=mean(df$PPV), PPV_025 = quantile(df$PPV, 0.025),

PPV_975 = quantile(df$PPV, 0.975),

nInf=mean(df$nInf), nInf_025=quantile(df$nInf, 0.025),

nInf_975=quantile(df$nInf, 0.975),

nTP = mean(df$nTP), nTP_025=quantile(df$nTP, 0.025),

nTP_975=quantile(df$nTP, 0.975),

nFP=mean(df$nFP), nFP_025=quantile(df$nFP, 0.025),

nFP_975=quantile(df$nFP, 0.975),

nTN=mean(df$nTN), nTN_025=quantile(df$nTN, 0.025),

nTN_975=quantile(df$nTN, 0.975),

nFN=mean(df$nFN), nFN_025=quantile(df$nFN, 0.025),

nFN_975=quantile(df$nFN, 0.975))

ov_dat=bind_rows(ov_dat, dat)

}

##write results as csv

write.csv(ov_dat, file = "young_22-2-22.csv")
